# Perspectives on Nurse Retention in Hospitals in the Netherlands: A Qualitative Study

**DOI:** 10.1155/2023/1390591

**Published:** 2023-12-28

**Authors:** Annamarike Seller-Boersma, Cecile Boot, Catharina van Oostveen, Irene Jongerden, Michele van Vugt

**Affiliations:** ^1^Amsterdam University Medical Centers, University of Amsterdam, Department of Human Resources, Amsterdam, Netherlands; ^2^Amsterdam Public Health Research Institute, Societal Participation and Health, Amsterdam, Netherlands; ^3^Amsterdam Public Health Research Institute, Quality of Care, Amsterdam, Netherlands; ^4^Amsterdam Research Center for Health Economics, University of Amsterdam, Amsterdam, Netherlands; ^5^Amsterdam University Medical Centers, VU University, Department of Public and Occupational Health, Amsterdam, Netherlands; ^6^Radboud University, Behavioural Science Institute, Nijmegen, Netherlands; ^7^Spaarne Gasthuis Academy, Spaarne Gasthuis Hospital, Hoofddorp, Netherlands; ^8^Erasmus University, Erasmus School for Health Policy and Management, Rotterdam, Netherlands; ^9^Amsterdam University Medical Centers, Department of Internal Medicine and Infectious Diseases, University of Amsterdam, Amsterdam, Netherlands; ^10^Amsterdam Public Health Research Institute, Global Health, Amsterdam Institute for Infection and Immunity, Amsterdam, Netherlands

## Abstract

**Aim:**

To explore hospital nurses' perceptions of working conditions that affect their retention and to identify preconditions for retention across nursing subgroups and informants directly involved in the work that nurses do.

**Introduction:**

Understanding why nurses want to stay in their job is essential for hospitals to improve retention and develop policies to combat nursing shortages. Retention barriers are known, but mostly pre-COVID-19 and in specific nursing subgroups, while nursing teams are diverse in life phase, education, and expertise.

**Materials and Methods:**

A qualitative study with semistructured focus group interviews with nurses from different hospitals. We held interviews with nursing students and with newly graduated, experienced, specialized, and master-educated nurses. In addition, we held interviews with informants directly involved in the work that nurses do.

**Results:**

Three themes concerning the perceptions of working conditions and retention preconditions were identified among subgroups: (1) nurses finding their personal pathway, indicating work that fits individual challenges during the life course and work that matches personal motives and authority and control over professional practice; (2) constructive collaboration within the nursing team and with their manager and physicians; and (3) availability of supportive facilities, e.g., development, professionalization, working environment, and rewards.

**Conclusion:**

Elements for retention occur at individual, team, and organizational levels. Nurses find it important that their profession aligns with their personal pathway and are motivated by constructive collaboration in a stimulating team. They emphasized organizational support in realizing career tracks and in active participation in decision-making. These themes are consistent across subgroups and encompass multiple interacting elements. *Implications for Nursing Management*. By recognizing and understanding what takes place at these different levels, policymakers and managers can develop effective strategies to promote nurse retention and improve healthcare quality. While implementing and monitoring a broad retention program, managers must remain attentive to nurses' perceptions of retention preconditions amidst changing demographics and the impact of COVID-19.

## 1. Introduction

Although today's nursing workforce is growing and the professional scope is expanding, this is insufficient to meet the rising demand for care [1]. Nowadays, patients are facing more and different forms of disorders and chronic conditions, possibly at the same time [2]. To cope with this changing healthcare demand, it is crucial to have enough well-trained nurses to provide safe and appropriate care.

According to the World Health Organization [1], there was a global shortage of nurses amounting to 5.9 million in 2018, which has been exacerbated by the COVID-19 pandemic. Nashwan et al. [3] report that nurses' intentions to leave their jobs were higher during the pandemic than before. The World Health Organization [4] predicts an increase in healthcare workers leaving their jobs due to COVID-19-related burnout, illness, and dissatisfaction. Shortage of nurses can result in poor outcomes for nurses, patients, and organizations, e.g., higher workload and work stress, lower quality of patient care, lower productivity, higher rates of absenteeism, and human capital depletion as intellectual capital exits the organization [5–7].

As a consequence, this can reduce the morale of the remaining staff [8]. Furthermore, nurse turnover is associated with an increase of costs [9, 10]. Turnover intentions play a crucial role in predicting the likelihood of nurses staying in their jobs [11]. Turnover intention is considered to be one of the stages in a complex decision-making process that results in turnover behavior [12]. Research shows that there are many factors related to retention of nurses, such as job satisfaction, work environment, work demands, social support, demographic factors, and opportunities to learn and grow [13–15]. Research more specifically focused on nurse retention in hospitals found factors like nursing management and leadership, personal influences, professional issues, staffing, relationships, appreciation, and financial remuneration [16, 17]. While it is known that all these different elements play a role, it is not known which of these elements hospital nurses consider as important from an overall perspective, including all relevant areas.

It is vital for hospital organizations to know what nurses themselves see as relevant, unambiguous elements for retention and to understand these reasons to detect early signs of nurses' intention to leave in order to develop retention strategies. However, current available studies mostly examine the perspective of one specific nursing subgroup, e.g., new graduate nurses [18], midcareer and older nurses [19], specialized nurses [20, 21], nurse educators [22], and nurse managers [23]. Yet, nursing teams tend to be diverse in formation, with variation in age, gender, and (additional) education. Furthermore, studies have been conducted from different perspectives, such as health [24], human resources, economics [25, 26], and management [27, 28]. Literature that explicitly includes what various nursing subgroups themselves believe is important for retention in hospitals is scarce and does not reveal differences between these subgroups. This knowledge is paramount for hospitals to develop policies to retain various types of nurses. In addition, many studies were conducted before the COVID-19 pandemic. Given the ongoing demographic changes and the impact of COVID-19, there is a need for new research in this area to enhance understanding of nurses' perspectives on retention within diverse nursing populations in the hospital. Examining multiple relevant subgroups in one study aids direct comparison and contributes to a more profound understanding of the factors influencing retention.

## 2. Aim

This study aims to explore how hospital nurses perceive their working conditions that affect their retention and to identify preconditions for retention across nursing subgroups and informants directly involved in the work that nurses do. The study findings will enhance our current knowledge of relevant elements of retention according to nurses with various backgrounds and levels of experience. This understanding will aid in the development of retention interventions in hospital settings and may guide nurses, managers, and policymakers to prevent nurses to actually leave the hospital.

## 3. Materials and Methods

We conducted a qualitative study using semistructured focus group interviews. Focus groups capture collective insights and interactions among diverse subgroups. We followed the four phases for focus groups outlined by Stewart and Shamdasani [29]: planning, recruitment, implementation, and analysis. Focus group interviews were held from February to April 2021. The reporting of this study complies with the consolidated criteria for reporting qualitative research (COREQ) recommendations [30].

### 3.1. Sampling and Recruitment

The study population comprises registered hospital nurses, nursing students, and informants directly involved in the work that nurses do. We added this latter group of participants due to their multifaceted, external perspectives on the nursing profession, from their roles in nursing advisory boards, management, research, and education. A small subgroup of informants consisted of physicians, human resource professionals, and business managers. Our sampling strategy is grounded in the recognition that nurses operate within a complex organizational system. Therefore, we consider informants as integral components of this system rather than isolating them as separate entities. Participants were purposefully recruited through an e-mail invitation distributed by contacts (mostly nurses of nursing advisory boards or nurse advisors) from academic hospitals, teaching hospitals, an employment agency, Universities of Applied Sciences, and a foundation that focuses on increasing the labor potential of nurses. When potential participants were willing to participate, they received an invitation for a focus group meeting as well as an information letter by mail with information about study background, privacy, and informed consent.

### 3.2. Data Collection

Semistructured focus group interviews were conducted between February and April 2021 by AS (MSc, background in social-cultural sciences), NR (MSc, background in quality and safety in healthcare), and JB (MSc, background in healthcare management), all former nurses with training in conducting interviews. The interviews were held online with Zoom (software) due to the COVID-19 situation. Participants were invited to a focus group interview that matched their background. We used homogeneous groups to ensure explicit attention to the perception of all nursing subgroups and avoid hierarchies in age or education level during focus group interviews: (1) nursing students and newly graduated nurses (<3 year after graduation), (2) experienced nurses (>3 year working as a nurse), (3) specialized nurses with supplementary training, (4) nurses with an additional master education (i.e., nursing science), and (5) informants directly involved in the work that nurses do; former nurses working in positions outside direct patient care, such as management, education, research, policy, or nurse advisory boards, added with physicians, human resource, and business managers.

At least two focus group interviews were organized per subgroup. Participants were required to have their video on during the call, and the recordings of the interviews were both video and audio. Each focus group interview lasted 90 minutes and started with the question: “What are your perceptions of working conditions that affect attractive work and retention of nurses in hospitals.” In the focus groups, moderators facilitated active engagement, creating a dynamic, inclusive atmosphere.

The moderators used an interview guide. This guide included auxiliary phrases to monitor that all respondents expressed themselves and general questions about nurses' perceptions on attractive work and retention, as well as specific preconditions for their own retention.

Prompts were used to explore participants' comments further during the interviews.

### 3.3. Data Analysis

Thematic analysis was used to identify common themes that motivate nurses to continue working in their hospital [31]. The analyses were discussed with the research groups who have extensive experience in qualitative research. The joint effort of the research team contributed to the study's credibility and dependability [32]. Of note, the researchers had no formal hierarchical relationship with the participants. The audio recordings of all focus groups were transcribed verbatim. First, three researchers (AS, NR, and JB) read and re-read the transcripts to get familiar with the data. Then transcripts were coded inductively. The first focus group was coded by all three researchers, and the interpretation of segments and codes was discussed until consensus was reached. Since consensus was high, the remaining focus groups were coded separately by two researchers (AS and NR or AS and JB) and then compared. During this comparison, the selected text segments and summary of the meaning of these text segments were reflected. A third researcher was consulted if there was no agreement. Once the initial codes were generated, the subsequent phase involved searching for themes. To accomplish this, the researchers (AS, NR, and JB) sorted the different codes into potential themes and linked all the relevant coded data extracts to the identified potential themes. Sticky notes were used to help sorting the different codes into potential themes. The phase ended with a collection of candidate themes and subthemes. The next phase consisted of reviewing the themes. Two levels of review and refinement were conducted for the candidate themes. The first level involved examining the coded data extracts, while the second level encompassed the entire data set. The fifth phase in the process, intended to define and name the themes, consisted of fine-tuning and making a more nuanced coding framework. The draft summary of the focus groups was then shared with all participants of the focus group interviews to enhance the credibility of the study [32]. Participants were asked if they recognized their perspective in the summary. Participants were also asked whether the content of the focus groups was correctly presented, whether things were missing, and whether the chosen quotes could be used anonymously.

### 3.4. Ethical Approval

The study was exempt from the Medical Ethical Review. Participation in the focus groups was voluntary. Each focus group started with consent of all participants for permission to record the interview. The transcripts of the interviews were stored and coded in accordance with the Dutch Personal Data Protection Act.

## 4. Results

### 4.1. Participants

Ninety-three participants from twelve different organizations within the Netherlands were present to join a focus group interview, and eight persons canceled their attendance or were absent. In total, thirteen semistructured focus group interviews were conducted (Table 1).

Participants ranged in age from eighteen to sixty-seven. Nine participants were male. There were seventy-four participants from academic hospitals, eight from teaching hospitals, eight from universities of applied sciences, one from an employment agency, and two from a foundation focused on enhancing the labor potential of nurses. Discussions were lively, with participants complementing one another, promoting the exploration of shared and differing viewpoints. This interaction yielded a rich exchange of experiences, albeit with variations in perceived importance.

### 4.2. Overview of the Findings

**Figure 1:**
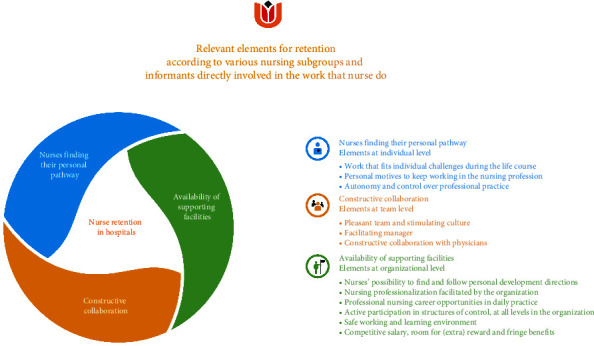
Relevant themes for retention in hospitals according to various nursing and informants directly involved in the work that nurse do.

Analysis of the interviews led to three main themes: nurses finding their personal pathway, constructive collaboration, and availability of supporting facilities. These themes comprise of twelve elements that are relevant for retention of hospital nurses (Figure 1).

The three main themes including twelve elements were identified by every subgroup of nurses and consistently present across all groups. Master-educated nurses and nurse-trained professionals who currently work (partly) outside of direct patient care provided more specific and detailed insights into the necessary preconditions and their perceptions of working conditions. In contrast, nursing students and newly graduated nurses mentioned fewer retention preconditions per element compared to the other groups.

#### 4.2.1. “Nurses Finding Their Personal Pathway,” Elements at Individual Level

Nurses highlighted that work that aligns with their personal career path is critical for retention. This included elements such as accommodating individual life course challenges, personal motivations to continue working in the nursing profession, and having autonomy and control over their professional practice.

*(1) Work That Fits Individual Challenges during the Life Course*. The significance of having work aligned with individual life challenges was emphasized by nurses. This included a schedule that accommodates the work-life balance, a manageable workload, emotional and physical well-being, and financial stability. In addition, the ability to adjust the formation or nurse-to-patient ratio for increased workload (e.g., due to task reallocation) or patient care complexity and intensity, as well as administrative tasks, was identified as crucial for retention. Nurses highlighted the necessity of schedule flexibility, self-scheduling, and regular work hours during certain life phases for retention. However, nurses experienced that these flexible options were increasingly limited due to staff shortages. A nurse with a master's degree from focus group 8 remarked:

*“We want to be embraced by the hospital at different life stages. We want to feel supported if you have children, that it is life stage dependent or situation dependent and therefore not “one size fits all.” So that the vitality of the nurse is considered.”*

*(2) Personal Motives to Keep Working in the Nursing Profession*. Nurses expressed their motivation to improve patient and family situations and contribute to the nursing profession. They strive to enhance the quality of life of their patients and offer support to patients and relatives during a vulnerable period like hospitalization. Nurses also seek opportunities to improve their nursing practice by sharing knowledge and experiences. They mentioned that they like to experience a challenging and versatile profession, which they described as working with different colleagues and disciplines to provide complex patient care. In the words of a clinical nurse specialist from focus group 8:

*“For me, nursing is really the most beautiful profession there is, it gives me all the opportunities to be able to really be there for the patient, and also the fact that as a nurse specialist, as a directing clinician, I can really do the process guidance of the patient, but also secure the patient journey by being next to the patient, and making sure that the patient moves through the healthcare system.” The next participant also identified with this sentiment, stating, “What's really beautiful about our profession is that we can make an incredible difference in people's lives during what are arguably the worst moments.”*

*(3) Autonomy and Control over Professional Practice*. Nurses emphasized the significance of autonomy and responsibility in carrying out daily tasks, care, and ancillary duties for retention. They also desired the possibility to be responsible for (the improvement of) the quality and organization of nursing work. Nurses wanted time to develop, share, and implement ideas for nursing care improvement at and around the bedside. Nurses noted the need for organizational agreements concerning time allocation for quality improvement and care organization development. This was perceived as a challenge, whereas in the current situation, where patient care cannot be postponed, priority is given to care delivery over development.

According to a nurse and PhD candidate participating in focus group 7:

*“For me, the very lack of autonomy in the nursing department was the reason I wanted to do something besides working as a nurse, because actually, I am fresh out of work, I have just been working for two years. The thing in doing research is that it enables me to decide things myself, have more responsibilities and figure things out on my own.”*

#### 4.2.2. “Constructive Collaboration,” Elements at Team Level

Nurses identified three essential elements for constructive collaboration that contribute to retention: a pleasant team and stimulating culture, facilitating manager, and constructive collaboration with physicians.

*(1) Pleasant Team and Stimulating Culture*. To ensure optimal patient care, nurses emphasized the importance of team engagement and collaboration. They referred to this as achieving equality within the team when carrying out tasks, valuing everyone's contributions, and appreciating each other's strengths and weaknesses. Nurses stressed the significance of personal development and having the ability to assume their respective roles within the team. They underlined the need for respect, equal treatment, and space for initiative and self-expression. Nurses accentuated the importance of a stimulating culture where all involved disciplines, including physicians, physiotherapists, and dieticians, support each other to deliver the best patient care and encourage each other's professional growth. From the perspective of an experienced nurse in focus group 8:

*“How nice it is to stand shoulder to shoulder together as a team, as the whole hospital, and all the nurses. That's ultimately the main reason you stay in your profession, I think.”*

This sentiment was echoed by a specialized nurse in focus group 6, who emphasized the importance of a stimulating culture:

*“Building a culture where professionalism is allowed to be there, where there is room for innovation, and where you also dare to stand out en speak up, without the manager or the team chopping your head off.”*

*(2) Facilitating Manager*. Nurses stated that a facilitating manager is vital for retention. They pointed out the importance of personal attention, individual support, and recognition from their manager. This included a manager who is accessible, knows what is happening, cooperates and intervenes when necessary, and is able to improve team cooperation. Nurses believed that a facilitating manager comprehends the essential task requirements, has a vision for nurses' development and training, and knows how to improve nursing care's quality and efficiency. The strength of a facilitating manager is demonstrated by his or her executive power: getting things done effectively. A newly graduated nurse in focus group 1 expressed:

*“What I really notice with us is that my manager keeps a very close eye on who likes what and who wants to do what. And so she also tries to organize a bit so that everyone gets his or her opportunities. Even if you haven't really thought about it yourself, she has suddenly thought of a project somewhere.”*

*(3) Constructive Collaboration with Physicians*. Nurses emphasized the importance of having approachable contact with physicians and their recognition of nurses' contributions to the quality of care. They indicated that recognition, acknowledgement, and exploitation of each other's roles and responsibilities are critical preconditions for constructive collaboration. Moreover, joint, multidisciplinary efforts to work on care improvement plans are also essential. Nurses reported that it contributes to their retention when physicians support their needs for professional development, through discussing ideas, providing guidance, and sharing their network and resources. A master-educated nurse from focus group 7 mentioned:

*“What also really helps is a strong network, setting up things with the medical discipline to improve care. Then you notice that things run faster, that there are more possibilities. That gives energy, that's together.”*

#### 4.2.3. “Availability of Supportive Facilities,” Elements at Organizational Level

According to nurses, the availability of supportive facilities for retention can be grouped into six relevant subthemes.

*(1) Nurses' Possibility to Find and Follow Personal Development Directions*. Nurses recognized the importance of personal and professional growth in their work environment, as it contributes to their overall job satisfaction and retention. They value organizations that encourage continuous learning and provide accessible training and development opportunities with support. This begins with an invitation to express their training and development needs, ensuring proper execution of their profession. In addition, nurses found it essential to have approachable access to development and training opportunities within the organization (e.g., participation in projects, attending conferences, clinical classes, training, courses, retraining, and follow-up courses). To enable such opportunities, nurses suggest that certain preconditions must be in place, such as facilitating development plans, availability of individual (training) budget and time and extension of opportunities to combine patient care and training (such as agreements on irregular shifts, planned development, and study time). An experienced nurse from focus group 3 articulated:

*“What keeps me in this hospital, despite the distance from home, are the development opportunities and the possibility to make use of an orientation internship at every ward for graduated nurses. It would be nice if there was a good overview of development directions and an annual personal budget, just like physicians. They can choose which congress or training they want every year.”*

*(2) Nursing Professionalization Facilitated by the Organization*. Nurses expressed the importance of working in hospitals that adhere to current protocols and guidelines as a standard. They require hospitals to organize the possibility to be and stay competent and skilled in the profession by education. Nurses stressed the need to collect data on the care provided, generate knowledge, and organize evidence to support nursing care when needed. Additionally, hospitals should allow sufficient time for nurses to deliver high-quality care and encourage and value nurses' improvement ideas and innovations. The availability of space and resources to continuously improve nursing care is also crucial, linked to an improvement cycle for implementing improvement ideas. A nurse director from focus group 13 stated:

*“We are a university hospital, education and research are definitely part of that, and that is pretty much underexposed among nurses. I think we should indicate that more strongly together. As a hospital, we have to live up to what we propagate.”*

*(3) Professional Nursing Career Opportunities in Daily Practice*. According to nurses, clear job descriptions that align with daily needs and practice are pivotal for retention. They highlighted the importance of facilitating professional growth in their career through support from managers, collaboration with physicians, and the provision of preconditions such as time and budget. Specifically, nurses with specialization and master training expressed a desire for varied job descriptions, particularly in daily practice, such as combining teaching or research with their nursing role. A nurse-trained professional currently working as business manager from focus group 12 expressed:

*“The highest a physician can achieve is to become a top specialist or professor, which is a job-related career prospect. The highest you can achieve now as a nurse is to take off your suit, leave the profession to become a full time lecturer, researcher, administrator or manager. Professional career opportunities I find seriously lacking for nurses, and that makes people say at some point, I've seen it now, let's do something else. Either outside care or within care. But at least no longer in the profession itself.”*

*(4) Active Participation in Structures of Control, at All Levels in the Organization*. To retain nurses, nurses emphasized the significance of receiving relevant information from the organization and having clear decision-making procedures in hospitals where nurses are involved.

Additionally, nurses stated that they want to be recognized as valuable partners in policy development and decision-making at the organizational level, including discussions on key developments and changes. To achieve this, nurses indicated that their opinions should be valued and incorporated into decision-making processes. They believe that by being able to influence others, feeling valued, and having the possibility to apply resources when needed, they can make a meaningful contribution to the success of the hospital. A nurse-trained professional currently working as manager from focus group 12 stated:

*“What is completely normal abroad, and abnormal in the Netherlands, is that the nursing line is mandatory in the leadership structure, right up to the board of directors.”*

*(5) Safe Working and Learning Environment*. A safe working environment is essential for nurses to perform their duties effectively and remain committed to their profession. According to nurses, this environment is established through open and transparent communication, where constructive feedback and accountability are encouraged. Mistakes must be accepted and treated as learning opportunities, while compliments should be given when due. A supportive learning climate should take into account differences in working styles, levels, ages, experiences, and cultural backgrounds. A climate that is fostered by colleagues who are open to everyone's input and who learn together. A nurse-trained professional currently working as lecturer at the University of Applied Sciences from focus group 9 remarked:

*“We train our students on the principle that you are at the steering wheel of your own development and that it is necessary to formulate your own learning goals for that purpose. When young graduates find themselves in an environment where you don't feel you can hold on to that enough, it's more than a disappointment. Former students tell me that it influences their intention to stay at that ward.”*

*(6) Competitive Salary, Room for (Extra) Reward, and Fringe Benefits*. Salary is considered a crucial element in retaining nurses, a salary that is on par with other sectors and healthcare professions with comparable responsibilities and educational levels. Nurses expressed that the current labor market has provided them with many opportunities, including the option to obtain an immediate permanent contract, training, extra salary, or other benefits elsewhere. To provide a sustainable solution, remuneration should take into account (extra) responsibilities, education levels, and years of experience, along with transparent employment conditions. In addition to regular salary, irregularity allowance, travel distance and costs, housing near the hospital, and vitality facilities (such as nutrition during night shifts, power nap benches, and eye protection) should also be taken into consideration. In the words of a nurse-trained professional currently working as lecturer at the University of Applied Sciences from focus group 9: “Nurses are service-oriented people, they never look that much at salary. But if you then compare what a nurse earns when she is bachelor-qualified with, say, bachelor-trained physiotherapists, dieticians, and occupational therapists graduates. Nurses start one or two salary scales lower. So you immediately feel put down then.” A nurse, part of the nurse advisory board, immediately replied: “I'd like to respond to this because I have reservations about whether salary truly serves as the main incentive for people to leave, especially among younger individuals. In my case, that is not the determining factor.”

## 5. Discussion

### 5.1. Key Findings

This qualitative study revealed three themes that influence how various nursing subgroups perceive their working conditions in the hospital in relation to retention and essential preconditions: how nurses can find their personal pathway, constructive collaboration, and the availability of supporting facilities. The main themes and elements were consistent across all nursing subgroups. Master-educated nurses and nurse-trained professionals provided more detailed insights, while nursing students and newly graduated nurses mentioned fewer retention preconditions per element. This is reasonable considering their stage of professional development and future aspirations.

The three main themes occur at multiple levels: individual, team, and organizational levels. By understanding what takes place at these different levels, policymakers and managers can develop effective strategies to promote nurse retention and improve healthcare quality.

Marufu et al. [16] also distinguish various domains that impact nurse retention. Chan et al. [13] recognize this, addressing nurse retention as a complex task because it requires interventions to occur at multiple levels. Although there is ample research on organizational factors and factors at the individual level, research on how the team level is associated with retention is scarce. In previous studies, the focus was primarily on individual and organizational levels [13, 16]. Certain elements, such as salary, are typically associated with the organizational level. It should be noted that in some countries, regulations and standards at the national or regional level govern the determination of these elements.

The three themes comprise of twelve elements that are relevant for the retention of hospital nurses. These elements are highly varied, encompassing individual fit, motivation, autonomy, teamwork, leadership, professional development, career opportunities, organizational support, participation, safety, and remuneration. Literature on nurse retention acknowledges the significant diversity of relevant elements [16, 17, 33]. During the focus group interviews, nurses mentioned several elements. De Vries et al. [33] state that retention factors are multifactorial and propose a model with intercorrelations derived from the considered retention literature on EU countries. This seems to demonstrate that factors influencing retention are closely interrelated and implies that implementing interventions on a particular theme or element can trigger consequential changes on other aspects as well. Despite the mention of this aspect by various nurses in the focus groups and its support in the literature, most studies only enumerate the relevant elements without demonstrating any correlation.

When exploring nurse retention from various perspectives such as themes, elements, and levels, the significance of nurses' autonomy and control becomes apparent. Ajzen [34] states that nurses' attitudes, subjective norms, and perceived behavioral control are the underlying factors influencing both intentions and actual behaviors. Self-efficacy, which reflects personal beliefs about one's ability to achieve specific goals, has been shown to reduce turnover intentions among nurses [35]. Literature supports the importance of nurses' autonomy, decision authority, control over tasks, and organization of the working day [36, 37]. In addition, extents to which working conditions provide nurses with opportunities for advancement and make full use of their skills and abilities [38]. Furthermore, organizational empowerment, a concept based on Kanter's structural empowerment model, is also positively associated with nurse retention. This includes employees' access to relevant information, support, and resources needed to perform their job, as well as opportunities to learn and grow [21, 39].

The results also demonstrate the significance of managers in the retention of nurses. Managers, and also policymakers, can potentially still influence intentions in order to prevent nurses from actually leaving the workforce [40]. Management support and appreciation play major roles in job satisfaction and retention [41–43], as managers have direct influence on nurses' development, professionalization, working environment, and rewards [20, 40]. In summary, the structural empowerment of nurses within the organization and the presence of supportive management are crucial factors that contribute to the retention of hospital nurses.

### 5.2. Implications for Nursing Management

When assessing the findings of this study, it is important for nurse managers and hospital leaders to recognize the themes identified as important for the retention of nursing subgroups. These themes are composed of multiple interacting elements that should be taken into account when designing policies or interventions to improve nurse retention and healthcare quality. Marufu et al. [16] also distinguish various domains that impact nurse retention and emphasizes that these factors can be influenced by different stakeholders, not only nurses and their employers. A broad range of interventions targeting these elements at individual, team, and organizational levels is necessary, considering that the impact of each intervention may vary. Structural organizational empowerment of nurses and supportive management are critical factors for implementing effective interventions. A retention program with diverse interventions targeting the identified subthemes at various organizational levels is necessary within hospitals. Implementation of these interventions can help to reduce the likelihood of nurses leaving their jobs, leading to positive effects such as enhanced quality of care, reduced financial costs, and improved morale among the remaining staff. To determine the effectiveness of the chosen interventions, it is essential to carefully monitor and evaluate the job satisfaction and retention of nurses in the hospital. Due to demographic shifts and the influence of COVID-19, managers must also stay attuned to nurses' perceptions of essential preconditions for retention.

### 5.3. Strengths and Limitations

A strength of our study was that we succeeded in including nurses from various subgroups. This diverse sampling strategy was implemented to ensure a broad acquisition of knowledge, reinforcing the reliability and trustworthiness of our findings. There was a great willingness to participate. Interviewed nurses indicated that they were pleased to contribute because the angle of enquiry was to find out what nurses themselves find important for retention. Each focus group interview was conducted with hospital nurses from only one subgroup. This was done deliberately to ensure explicit attention to the perception of all nursing subgroups and avoid hierarchies in age or education level during focus group interviews. Our approach enabled direct comparisons among multiple relevant subgroups and a profound understanding of the factors influencing retention. Participants recognized the findings, but discussion between groups might have led to different results. We did not organize focus group discussions with nurses who left the hospital as an indication of a failure in retention. Although this group may have additional viewpoints, our focus was on exploring elements that are important to motivate nurses to continue working in the hospital.

New research could examine nurses' perceptions of the critical or most relevant elements for retention, as well as identify which factors are lacking in specific hospital settings. Although all retention-related elements have been identified, further studies are needed to elucidate their interrelationships and potential synergy, which can inform the development and prioritization of effective interventions.

## 6. Conclusion

Our study has illuminated that nurse retention in hospitals is influenced by numerous factors that were recognized across multiple relevant subgroups. These factors encompass working conditions on individual, team, and organizational levels. Nurses find it important that their profession aligns with their personal pathway and are motivated by constructive collaboration in a stimulating team. They emphasized organizational support in realizing career tracks and in active participation in decision-making. These themes are consistent across all subgroups and encompass multiple interacting elements. It is important to devise a strategy and a retention program within the hospital targeting these themes. The challenge of managing nurse retention lies in the interplay of desired working conditions and the life phase of nursing subgroups. Meanwhile, managers, hospital leaders, and HR professionals must stay attentive to nurses' perceptions of essential retention preconditions throughout the implementation of this program, particularly in the context of demographic changes and the influence of COVID-19.

## Figures and Tables

**Table 1:** Number of participants per nursing subgroup.

Target group	Number of focus group interviews	Number of participants
Nursing students and newly graduated nurses	2	12
Experienced nurses	2	10
Specialized nurses with supplementary training	2	13
Master-educated nurses	2	18
Nurse-trained professionals currently working (partly) in positions outside direct patient care, such as management, education, research, policy, or nurse advisory boards (28), including physicians (2), human resource professionals (6), and business managers (4)	5	40
Total	13	93

## Data Availability

Due to the nature of this research, participants of this study did not agree for their personal data to be shared publicly, so supporting data are not available. Author elects not to share data.
